# An endophytic isolate of the fungus *Yarrowia lipolytica* produces metabolites that ameliorate the negative impact of salt stress on the physiology of maize

**DOI:** 10.1186/s12866-018-1374-6

**Published:** 2019-01-07

**Authors:** Farzana Gul Jan, Muhammad Hamayun, Anwar Hussain, Gul Jan, Amjad Iqbal, Aman Khan, In-Jung Lee

**Affiliations:** 1 Department of Botany, Garden Campus, Abdul Wali Khan University Mardan, Mardan, Pakistan; 2 Department of Agriculture, Garden Campus, Wali Khan University Mardan, Mardan, Pakistan; 3 Ministry of Education, Key Laboratory of Cell Activities and Stress Adaptations, School of Life Science, Lanzhou University China, Lanzhou, China; 4 School of Applied Biosciences, College of Agriculture and Life Sciences, Kyungpook National University, Daegu, South Korea

**Keywords:** *Y. lipolytica* FH1, Salt stress, Indole-3-acetic acid, Abscisic acid, Phenols, Flavonoids, Maize

## Abstract

**Background:**

To combat salinity, plants need easily accessible, safe and sustainable mechanisms for optimum growth. Recently, endophytes proved to be the promising candidates that helped the host plant to thrive under stress conditions. Therefore, the aim was to discover endophytic strain(s) and their mechanism of action to alleviate salt stress in maize.

**Results:**

Keeping the diverse role of endophytes in view, 9 endophytic fungi from the spines of *Euphorbia milli* L. were isolated. Among the isolated fungal isolates, isolate FH1 was selected for further study on the basis of high antioxidant activity and capability to produce high indole-3-acetic acid (IAA), indole-3-acetamide (IAM), phenol and flavonoid contents. The 18S rDNA sequence homology and phylogenetic analysis of the fungal isolate FH1 revealed to be *Yarrowia lipolytica*. Furthermore, the inoculation of *Y. lipolytica* FH1 had significantly promoted plant growth attributes in treated maize as compared to positive (salt stress) and negative (salt stress free) controls. Likewise, differences in chlorophyll, carotenes, electrolyte leakage, leaf relative water, peroxidase, catalase, ABA, IAA and proline contents were observed between treated maize and controls. Interestingly, *Y. lipolytica* FH1 inoculated plants showed lower endogenous ABA and higher endogenous IAA contents.

**Conclusion:**

From the results, we have concluded that *Y. lipolytica* inoculation has promoted the growth of maize plants through controlled metabolism and hormonal secretions (ABA and IAA) under salinity stress. Because of the fact, *Y. lipolytica* can be tried as an eco-friendly bio-fertilizer to achieve optimum crop productivity under saline conditions.

## Background

Maize (*Zea mays* L.) is an important cereal that contributes towards human food and animal feed worldwide [1]. The major maize producing countries include USA, Mexico, Nigeria, France, and Hungary. In Pakistan, maize is grown for the fresh market in various parts of Khyber Pakhtunkhwa. But the productivity of this valuable crop has been decreased over the years due to continuous change in the environment. Amongst various factors, salinity is one of the reasons that drastically affected the maize growth and productivity [2]. Salinity is in fact a global issue because more than 70 countries of the worlds have been marked by this problem. Also, rapid increases in the area of salt-affected land (45 million hectares in 1992 to 62 million ha in 2013) indicate the devastating nature of the problem. Based on this data, it is extrapolated that an area of 2000 ha catches salinity every day [3]. According to the statistical calculations, almost 6.5 million hectares of irrigated land in Pakistan is severely affected by various levels of salinity [4].

Interestingly, the most common source of salinity is irrigation water itself. Significant quantities of salts are contributed by using fresh water in irrigation, which was believed to have a negligible amount of salts. In dry hot areas, accumulation of salts is faster because of the rapid evaporation of irrigated water [3]. Climate change and high evaporation rate enhances salt accumulation in soil, which reduces soil water potential and water uptake by plant roots [5]. Prolonged exposures of plants to salinity results in excess accumulation of Na^+^ into plants, thus disturbing K^+^/Na^+^ balance and ultimately plant metabolism. Besides, plants exposed to salt stress can produce/accumulate excessive amounts of reactive oxygen species (ROS), disturbing electron transport chain in chloroplast and mitochondria [6]. Other consequences of hyper-accumulation of ROS, include membrane damage, cellular proteins, chlorophyll and nucleic acids damages [7].

To control production losses related to salinity in economically important crops, concrete biological measures are needed to optimize crop yield. Endophytic fungi residing in plant tissues can serve the purpose at the best [8]. Currently, utilization of endophytic fungi to mitigate severe salt stress in an environmental friendly way is an emerging technique to fix the problem. Endophytes can regulate plant physiological activities against abiotic stresses by releasing and/or enhancing the capability of the host plant to produce phytohormones (gibberellins, auxin, cytokinin, abscisic acid) and secondary metabolites (phenols and flavonoids) [9]. IAA is known to control root growth under salinity stress, inhibit H_2_O_2_ production, antagonize ethylene-based abscission and promote overall plant growth [10]. Flavonoids, on the other hand, can trigger IAA accumulation in the root nodules of host plants and motivate colonization of endophytes under stress conditions [8]. Similarly, ABA can control stomatal functioning to minimize water losses. Also, it can regulate stress-responsive genes to mediate plant defense system against stress damages [11]. Though there are studies related to the plant-microbe interaction, yet the field is still open to discover endophytes and their interactions with host plants under stress. In this study, we have focused on the role of endophytic *Y. lipolytica* FH1 in alleviating salt stress in maize seedlings.

## Results

### Isolation of FH1 from the spines of *Euphorbia milli*

From the surface sterilized spines of *Euphorbia milli* about 9 endophytic fungal strains were isolated. Furthermore, only the FH1 isolate have shown resistance to 100 mM NaCl salt stress and selected for further study.

### Physicochemical characteristics of FH1 strain

A 90% of fungal colonization frequency was observed on spine segments placed in Hagem media plates with FH1 (38%) was the most dominant colonizer (Table 1). Similarly, the isolate FH1 produced IAA, polyphenols and flavonoids in appreciable quantities and exhibited DPPH activity (Table 1).

**Table 1:** Potential of FH1 to colonize *E. milli* spine, exhibit DPHH activity and production of IAA, polyphenols and flavonoids

Fungal isolate	Colonization (%)	DPPH activity (%)	IAA (μg/ml)	Phenolics (μg/ml)	Flavonoids (μg/ml)
FH1	38 of 90	65 ± 8.52	9.17 ± 0.92	65.5 ± 3.95	10.6 ± 0.61

Colonization was measured as colonization frequency by plating spine segments on Hagem media plates at 28 °C for 7 days and counted the number of segments with endophytic colonies. About 90% of the segments were colonized by the 9 strains of endophytes, whereas 38% was colonized by FH1

### Molecular identification and phylogenetic analysis of FH1

**Fig. 1:**
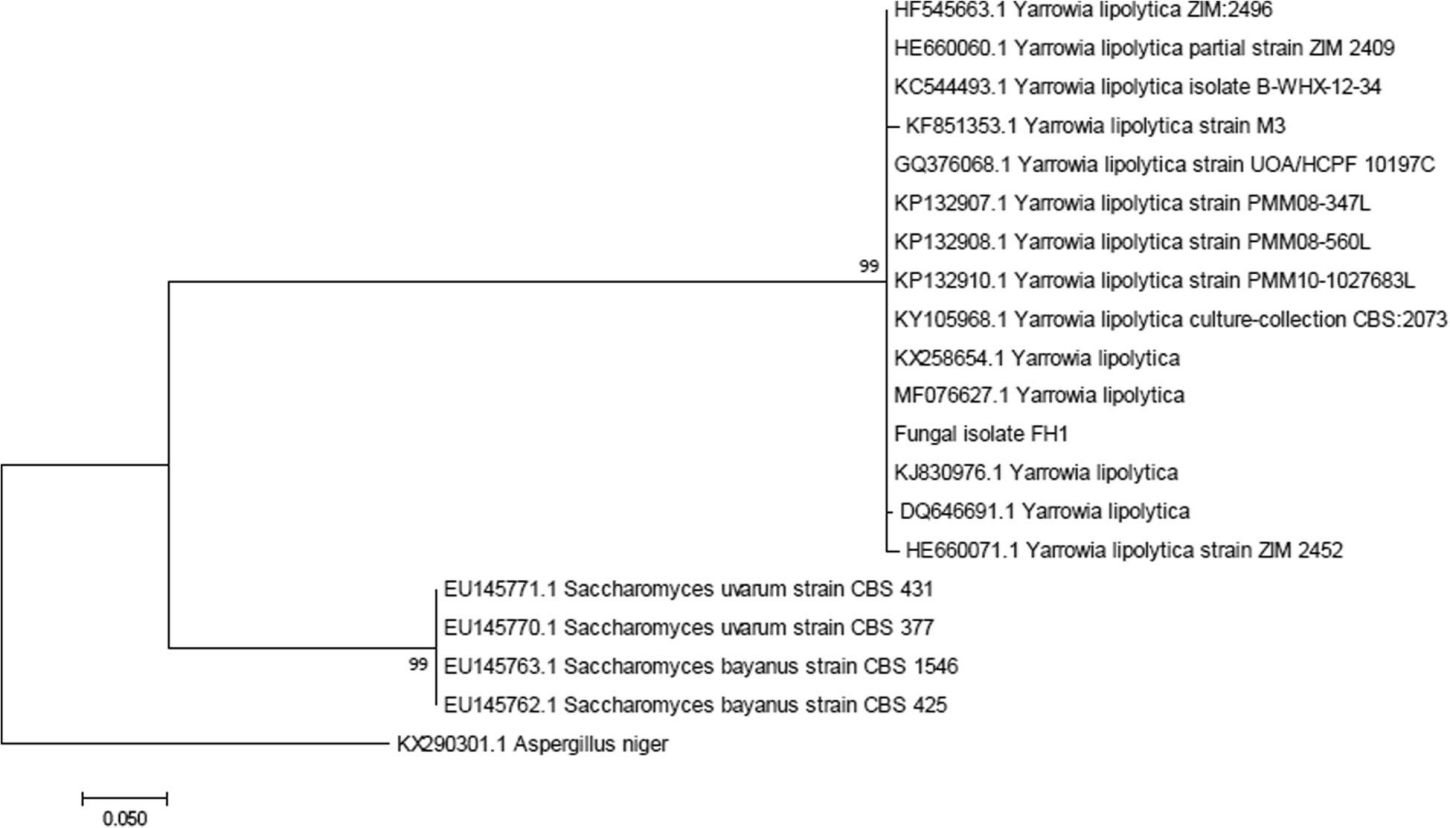
Identification of endophytic fungal isolate FH1 by phylogenetic analysis. The evolutionary history was inferred by using neighbor joining tree method based on the Tamura-Nei model. The analysis involved 17 nucleotide sequences. Evolutionary analyses were conducted in MEGA7. Bootstrap support of 57 for isolate FH1 with *Y. lipolytica* (99% sequence homology) strongly recommends our fungal isolate as *Y. lipolytica*

The isolate FH1 was identified by comparing its internal transcribed spacer (ITS) region including a partial sequence of 18S rDNA, the complete sequence of ITS1 and ITS2, the complete sequence of 5.8S rDNA and partial sequence of 28S rDNA genes sequence with the related sequences available in the GenBank database of NCBI (https://blast.ncbi.nlm.nih.gov/Blast.cgi). The isolate FH1 sequence showed 99% homology, 99% query coverage and 0.0 E values with 99% identity to *Yarrowia lipolytica*. The closely related sequences were retrieved from GenBank and subjected to phylogenetic analysis by using MEGA 7 to construct maximum likelihood tree. The isolate was grouped with *Yarrowia lipolytica* having 99 bootstrap supports (Fig. 1)*.* The gene sequence of the isolate FH1 was submitted to NCBI GenBank and was allotted with accession no. KY673731.

### Physicochemical alterations in Y*. lipolytica* inoculated maize plants under salt stress

#### Effect on chlorophyll and carotenoid contents of maize plants

**Fig. 2:**
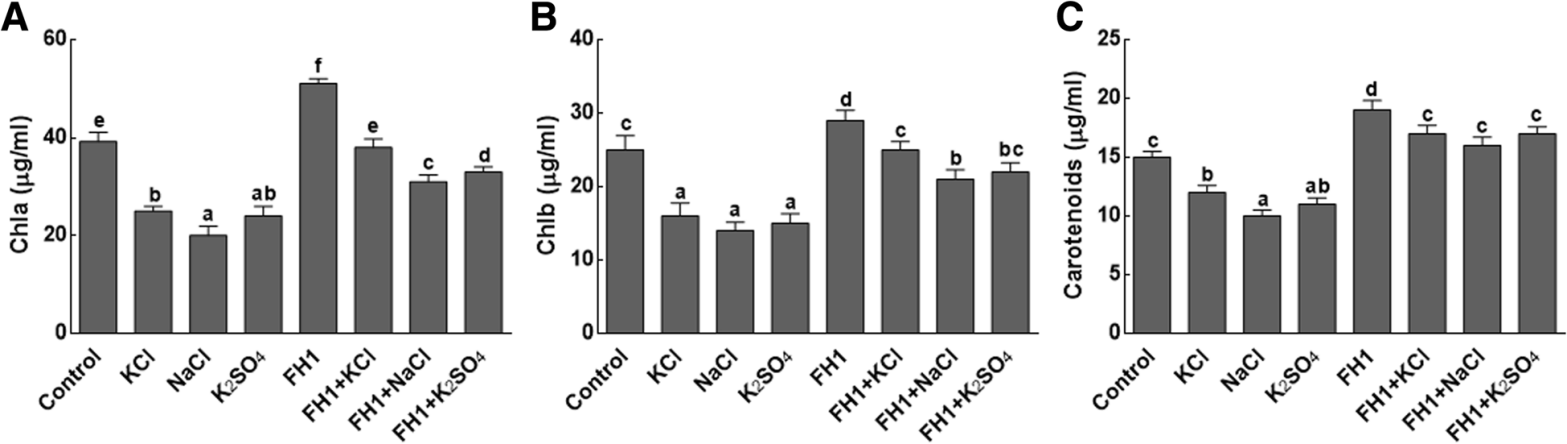
Effect of different salts on chlorophyll and carotenoids contents of maize plants. **a** represents chlorophyll a contents; **b** represents chlorophyll b contents; **c** represents carotenoids contents; Chla = chlorophyll a; Chlb = chlorophyll b; FH1 = fungal endophyte; KCl = potassium chloride; NaCl = sodium chloride; K_2_SO_4_ = potassium sulphate. Each bar represents the mean of triplicated data with ±SE. Bars that are labeled with different letters are significantly different from one another at *p* < 0.05

The fungal isolate FH1 greatly promoted the growth attributes of maize plants under salt stress, as the *Y. lipolytica* FH1 inoculated plants showed higher plant growth and pigmentation under stress conditions (Fig. 2). The fungal associated maize seedlings under salinity possessed significantly (*P* < 0.05) higher chlorophyll a (Fig. 2a), chlorophyll b (Fig. 2b) and carotenoid (Fig. 2c) contents compared to their respective controls.

#### Effect on growth parameters, electrolyte leakage, and relative water contents

**Fig. 3:**
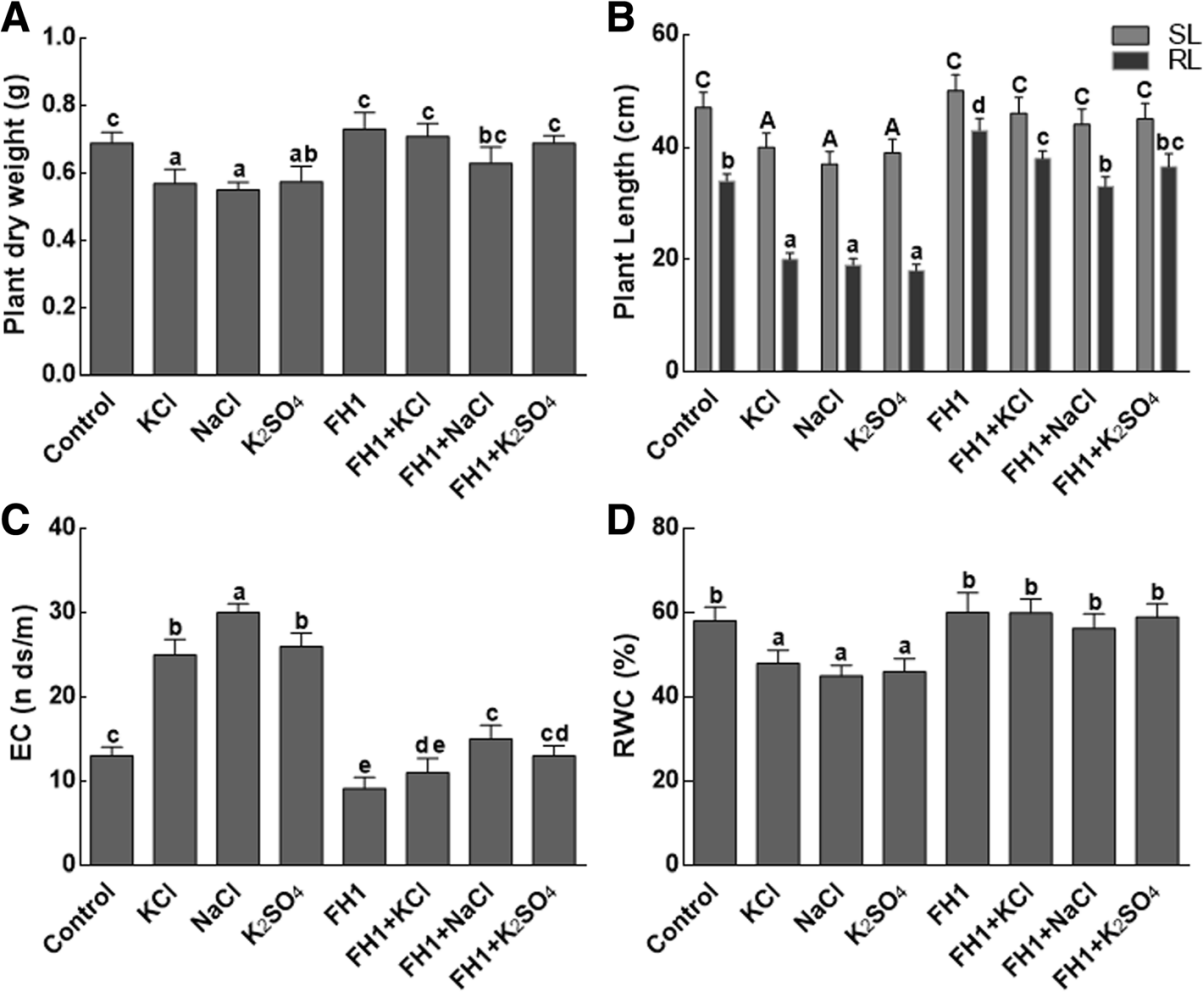
Effect of different salts on physiochemical characteristics of maize plants. **a** represents maize plant weights on dry and wet basis; **b** represents root and shoot lengths of maize plants under salt stress; **c** represents electrolytic leakage of maize plants under salt stress; **d** represents relative water content of maize plants under salt stress; DW = dry weight; SL = shoot length; RL = root length; EC = electrical conductivity; RWC = relative water content; FH1 = fungal endophyte; KCl = potassium chloride; NaCl = sodium chloride; K_2_SO_4_ = potassium sulphate. Each bar represents the mean of triplicated data with ±SE. Respective bars that are labeled with different letters is significantly different from one another at *p* < 0.05

From the results it is quite evident that the selected salts have significantly (*P* < 0.05) reduced plant weight as compared to the control (Fig. 3a). On the other hand, the salt stressed maize plants inoculated with *Y. lipolytica* FH1had lost less weight as compared to the control. A significantly (*P* < 0.05) higher weight have attained by the non-stressed, FH1 inoculated maize plants (Fig. 3a). Similarly, maize seedlings in association with FH1 have significantly longer roots and shoots as compared to the non-associated maize plants with or without salt stress (Fig. 3b).

Moreover, the electrolyte leakage was found significantly (P < 0.05) higher in salt stress treatments as compared to control (Fig. 3c). The NaCl (100 mM) triggered electrolytes leakage by 2.5 times higher, while the effect of KCl and K_2_SO_4_ (100 mM each) was 2 times as compared to the control. However, the fungal association has reduced the electrolyte leakage in maize seedlings under saline conditions (Fig. 3c). Likewise, a significant decrease in relative water content (RWC) of maize seedlings was observed in salt-stressed treatments (Fig. 3d). The RWC was higher in maize plants associated with *Y. lipolytica* FH1. A non-significant (*P* < 0.05) change has been observed in RWC of maize plants inoculated with FH1 as compared to the control plants (Fig. 3c).

#### Effect of *Y. lipolytica* FH1 on maize endogenous IAA and ABA contents

**Fig. 4:**
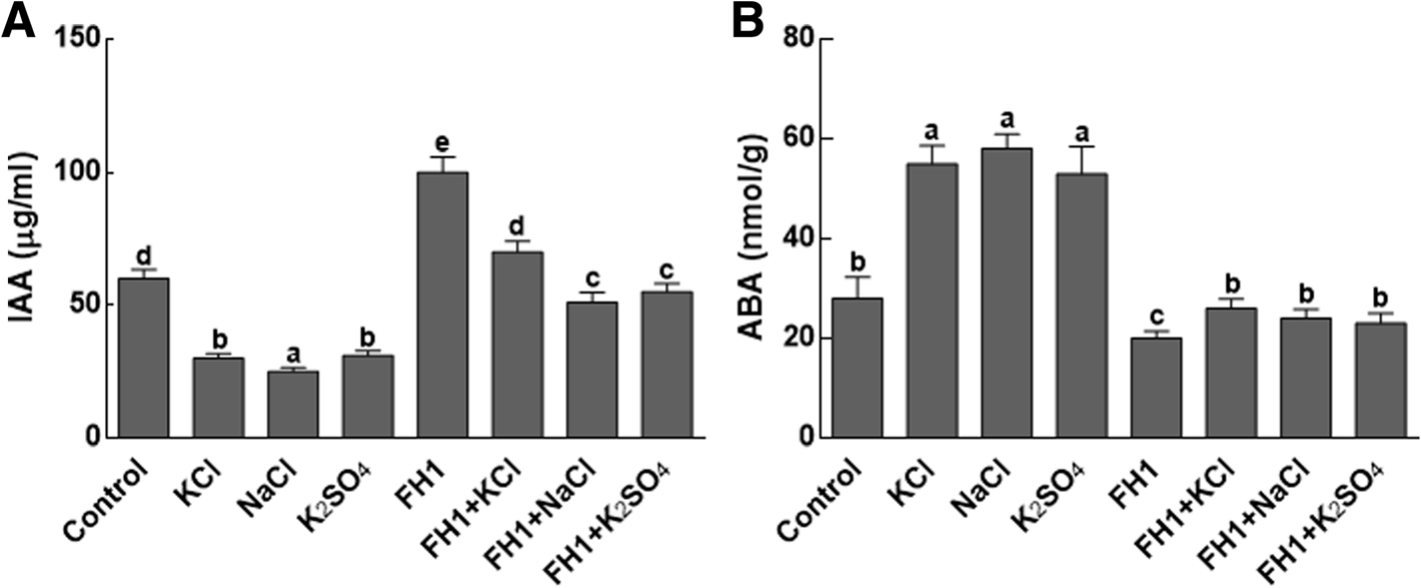
Quantitative changes in IAA and ABA contents of maize under salt stress. **a** represents IAA contents of maize plant under salt stress; **b** represents ABA contents of maize plants under salt stress; IAA = indole acetic acid; ABA = abscisic acid; FH1 = fungal endophyte; KCl = potassium chloride; NaCl = sodium chloride; K_2_SO_4_ = potassium sulphate. Each bar represents the mean of triplicated data with ±SE. Bars that are labeled with different letters are significantly different from one another at *p* < 0.05

The concentration of endogenous IAA was determined in endophytes associated and non-associated maize seedlings cultivated under control (no salt) and saline conditions. The results exhibited that the IAA contents of maize plants have increased by almost 100%, when the plants have inoculated with FH1as compared to the non-inoculated plants (Fig. 4a). A 3 fold increase in percent IAA contents has been recorded in FH1 inoculated stressed plants as compared to the salt stressed controls.

On the contrary, significantly (*P* < 0.05) higher amounts of abscisic acid (ABA) have been noticed in non-inoculated maize plants under salt stress as compared to the inoculated plants (Fig. 4b). Also, non-significant differences concerning the ABA contents have been noticed in the non-inoculated control treatments as compared to the inoculated plants under salinity. In short, the FH1 inoculated plants have significantly lower concentrations of ABA as compared to their respective controls (Fig. 4b).

#### Effect of *Y. lipolytica* FH1association on antioxidant enzymes system

**Fig. 5:**
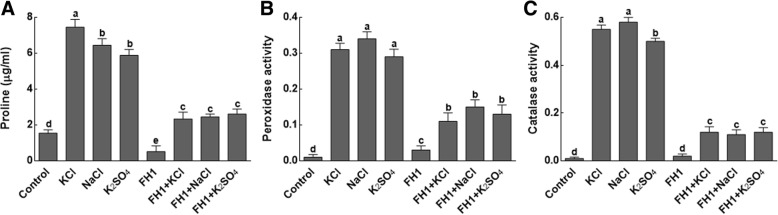
Effect of different salts sources on oxidizing capacity of maize plants. **a** represents phenolic contents of maize plants under salt stress; **b** represents peroxide activity of maize plants under salt stress; **c** represents catalase activity of maize plants under salt stress; FH1 = fungal endophyte; KCl = potassium chloride; NaCl = sodium chloride; K_2_SO_4_ = potassium sulphate. Each bar represents the mean of triplicated data with ±SE. Bars that are labeled with different letters are significantly different from one another at *p* < 0.05

Salt stress triggered much higher accumulation of proline, peroxidase (POD) and catalase as compared to the non-stressed plants (Fig. 5). However, the fungal inoculation of maize plants under salt stress has significantly (*P* < 0.05) controlled the production of proline, peroxidase (POD) and catalase. Approximately, 3-fold decrease in proline contents (Fig. 5a), 1.5-fold decrease in POD activity (Fig. 5b) and 5-fold decrease in CAT activity (Fig. 5c) have been noted in fungal inoculated maize plant as compared to non-inoculated plants.

## Discussion

Endophytic fungi depend on host plants for nourishment, water and physical protection against biotic and abiotic adversities. In return, the endophyte secretes secondary metabolites, e.g. phenols and flavonoids [12]. A wide array of biologically active secondary metabolites has been isolated from endophytic fungi [13, 14]. Nonetheless, very little knowledge is available regarding the synthesis and secretion of plant growth regulators, phenols, flavonoids, DPPH activity of the endophytic fungi culture filtrate under salt stress. In this study, we isolated 9 endophytic fungi from the spines of *E. milli*, a well-known xerophyte. *Y. lipolytica* FH1 was selected for current study based on its resistance to 100 mM salt stress and IAA biosynthesis capabilities. The CF of *Y. lipolytica* FH1 was screened for secondary metabolites. *Y. lipolytica* FH1 produced IAA and promoted growth of maize seedlings. Therefore, restoration of growth in treated maize seedlings might be attributed to the IAA secreted by FH1. The ability of endophytes to produce IAA can be linked with two important putative genes (IaaM and IaaH). The putative IaaM and IaaH genes code for enzymes tryptophan-2-monooxygenase and indole-3-acetamide hydrolase suggested that IAA biosynthesis was a two-step process that progresses via an indole-3-acetamide pathway [15]. Waqas et al. (2012) reported the detection of IAA in the culture filtrates of endophytic isolates of *P. glomerata* and the positive role of IAA in plant growth promotion under saline conditions. Our current findings regarding IAA production and mitigation of salt stress by *Y. lipolytica* FH1 are in close agreement with that of N Saeed, MR Khan and M Shabbir [16].

On the other hand, various reasons are responsible for the decrease in crop growth under salinity. One of the important reasons may be the limitation of photosynthesis in plants exposed to saline conditions. Under salinity stress chloroplast accumulates excessive Na^+^, which inhibits PS-II activity and thus deters photosynthesis and ultimately growth rate [17]. Also high salinity induces ethylene production in plants, which inhibit chlorophyll biosynthetic pathway [18]. High amount of Na^+^ in the maize cell might hinder the growth and development of maize through negative osmotic potential, thereby reducing the uptake of water and minerals like K^+^ under salinity stress. The response of maize seedlings to selected salts (KCl, NaCl and K_2_SO_4_) in the current study was different. Among the selected salts, NaCl was the most toxic that limited the growth of maize by interfering with the physiological processes of the maize. The adverse effects of NaCl were evident from low chlorophyll contents, reduced shoot and root growth, lighter plant (fresh and dry weight basis), low relative water contents and higher electrolytes leakage. The decrease in plant relative water content reflects on physiological drought forcibly imposed on plants, as soil solution in saline areas is hypertonic in relation to cell sap [8].

Another phenomenon that was affected by salt stress was changes in endogenous phytohormones, i.e. high ABA and low IAA content. ABA is reported to perform key role in the regulation of signaling pathways, which are involved in plant growth and development under salt stress [19]. It has previously been demonstrated that fungal inoculation has a role in increasing ABA contents of host plant under salt stress [13]. Similarly, IAA is essential for plants growth and development due to its function in root, axillary bud and flower development. Certainly, IAA (either exogenous or endogenous) can regulate various developmental processes in plants under abiotic stress [20, 21]. In this study, high contents of IAA were noticed in plants that were inoculated with *Y. lipolytica* FH1 and exposed to salt stress. Our results suggested that ABA and IAA have contrasting roles in plant response to salt stress.

Additionally, the inoculated *Y. lipolytica* FH1 helped the maize seedlings to stand against salt stress that was quite evident from the accumulation of pigments (chlorophyll and carotenoids content), a decrease in electrolyte leakage, increased seedlings growth and improvement in leaf relative water content. Indeed, maize seedlings tried to withstand the adverse effects of salts by increasing production of the POD, catalase, and proline. The results suggested that under stress conditions, endophytic fungi modulated the host plant system in order to detoxify the hazardous ROS rather enabling the host plant to avoid uptake of salts. Scavenging of the salt-induced H_2_O_2_ by catalase and peroxidase may be an effective mechanism of plants to overcome salinity stress [22]. Significant aggregation of proline in *Paecilomyces formosus* associated plants growing under salinity stress, suggesting a decline in ionic influx inside the cellular masses and rescuing plants to maintain its osmotic balance [16]. Besides, the up-regulation of genes associated with CAT, POD and proline in maize plants, the endophyte might also contribute towards the resistance against salinity stress [23] which supports our results.

## Conclusion

From the current findings, we have concluded that the endophytic association of *Y. lipolytica* FH1 with maize plants has significantly improved the growth of maize under salt stress. The recovery of fungal inoculated maize plants (in terms of growth and development) under stress conditions can be linked with the secreation of exogenous IAA and regulation of maize endogenous IAA and ABA. In fact, the normal secretion of endogenous IAA and ABA by maize plants under salinity can be attributed to the association of *Y. lipolytica* FH1. Also, FH1association has controlled the production of POD, CAT and proline in maize plants under salt stress.

## Methods

### Isolation of endophytic Fungi

In the present study, 9 endophytic fungal isolates were isolated from the spines of *Euphorbia milli*, an ornamental plant easily available at Abdul Wali Khan University, district Mardan. The district lies from 34° 05′ to 34° 32′ north latitudes and 71″ 48′ to 72° 25′ east longitudes. The average temperature of Mardan is 23.2 °C with an average rain fall of 110 mm. The characteristics of the soil ranges from sandy loam to clay with pH from 5.9 to 8.1.

Initially, the spines from the collected plants were cut and washed with tap water. Surface sterilization was carried with 70% ethanol for 1 min. The sterilized spines were then washed with double distilled water (ddH_2_O) to remove traces of ethanol. To isolate endophytic fungi, the spines were cut into 0.5 cm segments and placed carefully in Petri-plates containing Hagem growth media under conditions mentioned above. To confirm the effectiveness of 70% ethanol, the spines were cut into segments and were surface sterilized. The sterilized pieces of spines were then placed on Hagem media [24] and processed as mentioned earlier. The fungal isolates collected from the Hagem media were grown on PDA media [25].

Endophytic fungi were isolated on Hagem media plates incubated at 28 °C for 7 days. Subculturing of the endophytic fungal isolates was performed by growing the isolates on PDA plates incubated at 28 °C for 7 days. For secondary metabolites production, the isolated endophytes were grown in Czapek broth (Peptone 1%, Glucose, 1, 0.05% MgSO_4._7H_2_SO_4,_ 0.05% KCl and 0.001% FeSO_4._7H_2_SO_4_ pH 7.3 ± 0.2) maintained at 28 °C and 120 rpm for 7 days [26]. After incubation, fungal cultures were filtered to obtain fungal biomass and respective culture filtrate (CF).

### Colonization frequency

Colonization was measured as colonization frequency by plating spine segments on Hagem media plates at 28 °C for 7 days and counted the number of segments with FH1colonies. The colonization frequency of FH1was then calculated as following:

$$ \% Colonization\ frequency=\frac{Total\ twig\ section\ colonized}{Total\ number\ of\ segments\ colonized}\times 100 $$

### Salt stress tolerance

Fungal isolate was taken from PDA plates and inoculated in 50 ml Czapek-broth supplemented with 100 mM NaCl to check its ability to resist salt stress. The cultures in the flasks were then incubated for 7 days at 28 °C and at 120 rpm. After 7 days of incubation, the growth of the inoculated fungal isolates was observed. The endophyte FH1 was selected for further study based on its ability to resist 100 mM NaCl.

### Determination of IAA in culture filtrates of FH1 through GC/MS

Fungal culture filtrate obtained as described earlier were subjected to gas chromatography-mass spectroscopy (GC/MS-SIM) coupled with selected ion monitoring [27]. The CF of isolate was taken in test tubes and filtered through 0.45 μm cellulose acetate filters. The pH of filtrate was adjusted to 3.0 with 1 N HCl and then extracted with ethyl acetate. The organic layer was evaporated at 45 °C in a water bath. The dried sample was then dissolved in 5 ml of 0.1 M acetic acid and eluted through the reverse phase C18 column using 30, 50, 100% methanol. Methanol from the eluted samples was evaporated at 45 °C using a water bath. The residues left were dissolved in 1 ml of methanol and added 1.5 ml of ethereal diazomethane to prepare methyl ester fraction. The methylated samples were re-dissolved in ethyl acetate before being analyzed by GC/MS-SIM (6890 N network GC system, and 5973 network mass selective detector; Agilent, Palo Alto, CA, USA). For IAA, 1 μL of the sample was injected in a 30 m × 0.25 mm i.d., 0.25 μm film thickness DB-1 capillary column (J & W Scientific Co., Folsom, CA, USA), respectively. The GC oven temperature was programmed as: holding temperature of 70 °C for 2 min, then rise to 200 °C (with a steady increase in temperature, i.e. 20 °C/ minutes) and finally reached to 285 °C (with an increase of 5 °C/ minutes). Helium as a carrier gas was maintained at a head pressure of 30 kPa. The GC was directly interfaced to a mass selective detector with an interface and source temperature of 230 °C, an ionizing voltage of 70 eV, and a dwell time of 100 min.

### Total phenolics determination

The total phenolics were determined in the CF of isolate FH1 following the method proposed by A Qawasmeh, HK Obied, A Raman and W Wheatley [28]. A blue coloration indicates the presence of phenol and their absorbance was checked at 650 nm using SHIMADZU spectrophotometer (Kyoto, Japan). Gallic acid was used as a standard to quantify the total phenol in CF of FH1.

### Total flavonoids determination

Colorimetric determination of flavonoids was carried out in the CF of isolate FH1, following the protocol of K Srinivasan, L Jagadish, R Shenbhagaraman and J Muthumary [29]. Quercitin was used as a standard for flavonoids determination. A white milky coloration indicates the presence of flavonoids.

### 2, 2 Diphenyl-1-Picrylhydrazyl (DPPH) % activity

The CF obtained from our isolate FH1 grown in Czapek medium was assayed for scavenging of the reactive oxygen species (ROS) according to Liu et al., (2007). Approximately, 2 mL of distilled water, 1 mL of 0.1 mM 2, 2 Diphenyl-1-picrylhydrazyl (DPPH) was added to 0.5 mL of CF from isolate FH1. The mixture was shaken vigorously and allowed to stand for 30 min at room temperature. The antioxidant activity of CF was determined by measuring the decrease in absorbance at 517 nm against blank. Methanol was used as a blank and ascorbic acid was used as positive control.

### Fungal identification and phylogenetic analysis

A molecular approach was adopted to identify the selected fungal isolate FH1. The genomic DNA of isolate FH1 was extracted following the established protocol [30] which was previously modified [31]. The fungal isolate FH1 was identified by sequencing the internal transcribed spacer (ITS) region of 18S rDNA using universal primers ITS-1 and ITS-4 [32]. The sequence obtained was subjected to BLASTn1 for sequence homology estimation. The evolutionary history of sequences obtained as a result of homology search was inferred by using the Maximum Likelihood method based on the Tamura-Nei model [33]. The analysis involved 25 nucleotide sequences and the evolutionary analyses were conducted in MEGA7 [34]. The gene sequence of the isolate FH1 was submitted to NCBI GenBank and was allotted with accession no. KY673731.

### Plant growth promotion and salt stress alleviation potential of FH1

Healthy and uniform seeds (30 seeds) of *Zea mays* L. were surface sterilized by dipping them in 0.1% HgCl_2_ followed by washing with double distilled water. Seeds were then allowed to germinate in Petri plates on wet filter papers at 26 ± 1 °C in order to get equally germinated seedlings. After germination, seedlings were transferred to pots (pot size width 9 cm/length 11.3 cm) containing autoclaved soil (texture = clay loam; pH = 7.21; EC = 2.1) and the seedlings were allowed to grow and establish in soil (for 2-days) before fungal biomass was added to the soil. Soil inoculation was performed by drench inoculation method that involved the application of fresh biomass of selected fungal strain FH1 (0.3 g/ 300 g soil) around the root zone in order to assess their ability of alleviating salinity stress in maize [35]. Salinity stress of 25 ml of water containing 100 mM NaCl or KCl or K_2_SO_4_ was applied after every three days for 20 days. The pots have no pores to allow leakage of water and salts. The plants were watered daily. The experiment was terminated after completion of salt stress and plant growth attributes were analyzed, following the established protocol [36]. The plant dry biomass was measured after drying the plants at 70 °C for 48 h in an oven [37]. Contents of Chl a, Chl b, and carotenoids in the extract of fully expanded maize leaves before harvest was determined using MacKinney equations [38]. Fully expanded fresh leaves of fungal inoculated and non-inoculated maize plants under salt stress were homogenized with 2 ml of acetone (80%) and washed twice to reach final volume of 7 ml. The absorbance was measured using a spectrophotometer at 480 nm, 645 nm, and 663 nm. Electrolyte leakage was determined by adopting standard method [39]. Leaf relative water contents (%) was measured by following the standard protocol [40]. For the determination of POD, CAT, proline, IAA and ABA, maize seedlings were cut into pieces and frozen immediately in liquid nitrogen and stored in freezer at − 70 °C. The pot experiment was done in triplicate with an experimental outline as follows:

1. Control plants (no salt stress + no fungal inoculation)
2. NaCl treated plants (100 mM NaCl + no fungal inoculation)
3. KCl treated plants (100 mM KCl + no fungal inoculation)
4. K_2_SO_4_ treated (100 mM K_2_SO_4_ + no fungal inoculation)
5. FH1 inoculated treatment (no salt stress)
6. NaCl treated inoculated plants (100 mM NaCl + FH1inoculation)
7. KCl treated inoculated plants (100 mM KCl + FH1inoculation)
8. K_2_SO_4_ treated inoculated plants (100 mM K_2_SO_4_ + FH1inoculation)

### Quantification of maize plant endogenous IAA and ABA

Plant hormones extraction and purification were analyzed by using HPLC according to the method of Kettner and Döerffling [41]. Fresh plant leaves (1 g) were ground at 4 °C in 80% methanol with butylated hydroxy toluene (BHT) as an antioxidant. After 72 h extraction, the pooled extract was centrifuged at 3000 rpm and the supernatant was partitioned at pH 2.5–3 with ethylacetate (1/4th volume of the extract). The ethyl acetate phase was dried down completely on the rotary thin film evaporator (RFE) and the residues were re-dissolved in 100% methanol. The samples were then passed through a Millipore filter (0.45u) and were analyzed by HPLC (Agilent 1100), equipped with variable UV detector and C18 column (39 × 300 mm) (BondaPack Porasil C18, 37/50 μm, Waters, Eschborn, BRD). Methanol and water in the ratio of (30:70; *v*/v) were used as mobile phase at a flow rate of 1500 μL/min and a run time of 20 min/sample. The plant hormones were identified on the basis of retention time of phytohormone standards.

### Determination of catalase activity

Catalase activity was assayed by previously described method [42]. The activity was estimated by a decrease in the absorbance of H_2_O_2_ at 240 nm (1 U of catalase was defined as conversion of 1 μmole of H_2_O_2_ per minute).

### Estimation of peroxidase activity

Peroxidase activity (POD) was assayed by following established protocol [43]. Added, 5.55 g of CaCl_2_ to 100 ml of distilled H_2_O_2_ and the resultant solution was cooled on ice prior to use in the assay. After cooling, mixed, 0.5 ml p-Phenylenediamine, 1.5 ml MES, 0.1 ml plant extract, and 0.45 ml H_2_O_2_. The MES Buffer alone was used as blank and the optical density was measured at 510 nm, initially soon after mixing the contents and then after 3 min of incubation at room temperature.

### Proline contents

The proline contents of maize seedling was measured according to the method described by JJ Shaw, DJ Spakowicz, RS Dalal, JH Davis, NA Lehr, BF Dunican, EA Orellana, A Narváez-Trujillo and SA Strobel [44]. The fresh leaf samples (0.2 g) were homogenized in 5 ml (3%) of aqueous sulphosalicylic acid and the homogenate was subjected to centrifugation at 12,000 rpm for 10 min. Equal volume of glacial acetic acid ninhydrin was then added to the supernatant. The mixture was boiled on a water bath adjusted at 100 °C for 1 h and then extraction was done with 4 ml of toluene. The absorbance was measured at 520 nm using toluene as a blank.

### Statistical analysis

Data analysis was done using SPSS 20 for Windows. Means comparison was done by analysis of variance (ANOVA) and Duncan multiple range test at *P* = 0.05.

## Abbreviations

ABA: Abscise acid
CAT: Catalases
CF: Culture filtrate
DNA: Deoxyribo nucleic acid
DPPH: Diphenyl-1-Picrylhydrazyl
GC: Gas chromatography
GC/MS-SIM: Gas chromatography/mass spectroscopy- selected ion monitoring
HPLC: High performance liquid chromatography
IAA: Indole-3-acetic acid
POD: Peroxidases
